# Adapting Problem Management Plus for Implementation: Lessons Learned from Public Sector Settings Across Rwanda, Peru, Mexico and Malawi

**Published:** 2021-03-31

**Authors:** Sarah F. Coleman, Hildegarde Mukasakindi, Alexandra L. Rose, Jerome T. Galea, Beatha Nyirandagijimana, Janvier Hakizimana, Robert Bienvenue, Priya Kundu, Eugenie Uwimana, Anathalie Uwamwezi, Carmen Contreras, Fátima G. Rodriguez-Cuevas, Jimena Maza, Todd Ruderman, Emilia Connolly, Mark Chalamanda, Waste Kayira, Kingsley Kazoole, Ksakrad K. Kelly, Jesse H. Wilson, Amruta A. Houde, Elizabeth B. Magill, Giuseppe J. Raviola, Stephanie L. Smith

**Affiliations:** 1 Partners In Health, Boston, USA; 2 Partners In Health/Inshuti Mu Buzima, Rwanda; 3 Department of Psychology, University of Maryland, College Park, USA; 4 School of Social Work and College of Public Health, University of South Florida, Tampa, USA; Department of Global Health and Social Medicine, Harvard Medical School, Boston, USA; 5 Partners In Health/Inshuti Mu Buzima, Rwanda; 6 Partners In Health/Inshuti Mu Buzima, Rwanda; 7 Partners In Health/Inshuti Mu Buzima, Rwanda; 8 Partners In Health/Inshuti Mu Buzima, Rwanda; 9 Partners In Health/Socios En Salud, Peru, Harvard Global Health Institute, Boston, USA; 10 Partners In Health/Compañeros En Salud, Mexico; 11 London School of Hygiene & Tropical Medicine; 12 Partners In Health/Abwenzi Pa Za Umoyo, Malawi; 13 Partners In Health/Abwenzi Pa Za Umoyo, Malawi; Division of Pediatrics and Division of Hospital Medicine, Cincinnati Children’s Hospital Medical Center, Cincinnati, OH, USA; 14 Partners In Health/Abwenzi Pa Za Umoyo, Malawi; 15 Partners In Health/Abwenzi Pa Za Umoyo, Malawi; 16 Partners In Health/Abwenzi Pa Za Umoyo, Malawi; 17 Partners In Health, Boston, USA; 18 Partners In Health, Boston, USA; 19 Partners In Health, Boston, USA; 20 Icahn School of Medicine at Mount Sinai, New York, USA; 21 Partners In Health, Department of Global Health and Social Medicine, Harvard Medical School, Boston, USA; The Chester M. Pierce, MD Division of Global Psychiatry, Massachusetts General Hospital, Boston, USA; 22 Partners In Health, Boston, MA; Department of Global Health and Social Medicine, Harvard Medical School, Boston, USA; Department of Psychiatry, Brigham and Women’s Hospital, Boston, USA

**Keywords:** common mental health conditions, curriculum adaptation, public sector, Problem Management Plus (PM+), task-sharing

## Abstract

Problem Management Plus (PM+) is a low-intensity psychological intervention developed by the World Health Organization that can be delivered by nonspecialists to address common mental health conditions in people affected by adversity. Emerging evidence demonstrates the efficacy of PM+ across a range of settings. However, the published literature rarely documents the adaptation processes for psychological interventions to context or culture, including curriculum or implementation adaptations. Practical guidance for adapting PM+ to context while maintaining fidelity to core psychological elements is essential for mental health implementers to enable replication and scale. This paper describes the process of contextually adapting PM+ for implementation in Rwanda, Peru, Mexico and Malawi undertaken by the international nongovernmental organization Partners In Health. To our knowledge, this initiative is among the first to adapt PM+ for routine delivery across multiple public sector primary care and community settings in partnership with Ministries of Health. Lessons learned contribute to a broader understanding of effective processes for adapting low-intensity psychological interventions to real-world contexts.

## Introduction

Mental health conditions contribute to a substantial burden of disease, accounting for almost a third of years lived with disability worldwide and depression affecting more than 300 million people globally (Jacob & Patel, 2014; Vigo et al., 2016). Effective evidence-based interventions such as cognitive behavioural therapy are available in some low- and middle-income countries. However, there is up to a 90% treatment gap, as they historically require trained mental health specialists making them more costly and lengthy (Patel et al., 2010).

Problem Management Plus (PM+), first published in 2016, is a brief, low-intensity transdiagnostic psychological intervention developed by the World Health Organization (WHO) to address mental health treatment gaps in low- and middle-income countries (WHO, 2016). PM+ enables nonspecialist or lay health providers to address common mental health conditions for people living in adversity, teaching four primary strategies across five sessions: (1) stress management, (2) problem solving, (3) behavioural activation and (4) strengthening social support, as well as relapse prevention. Emerging global evidence demonstrates the efficacy of PM+ to reduce psychological distress when delivered to individuals or groups (Dawson et al., 2016; Perera et al., 2020; Sangraula et al., 2020).

Successful implementation of PM+ and other psychological interventions require contextual and cultural adaptation to increase treatment acceptability, user satisfaction and effectiveness (WHO, 2016). However, published literature rarely describes the cross-site adaptation processes for psychological interventions to context or culture, including curriculum or implementation adaptations (Chowdhary et al., 2014). Practical guidance on PM+ adaptation for use in real-world settings while maintaining fidelity to core psychological elements of PM+ is essential for mental health implementers to enable replication and scale globally.

This paper describes the cross-site process of adapting PM+ for implementation in community and primary care settings in Rwanda, Peru, Mexico and Malawi undertaken by the nonprofit organisation Partners In Health (PIH) between 2016 and 2020. To our knowledge, this is among the first initiatives to adapt PM+ in partnership with Ministries of Health (MoH) for routine care delivery in public sector settings outside of research or emergency response. We summarise the sequential adaptation of PM+ to context beginning with Rwanda, leveraging our understanding of necessary considerations for PM+ implementation across multiple sites. Given the breadth of this work, we aim to share lessons learned that can contribute to a broader understanding of effective processes for cross-site adaption of low-intensity psychological interventions to real-world contexts. Detailed site-specific papers describing the local adaptation of psychological interventions will follow, starting with a field report of PM+ adaptation in Mexico (Rodríguez-Cuevas et al., 2021).

## Methods

### Cross-Site Setting

PIH is an international nongovernmental organisation that promotes health systems strengthening in close collaboration with government MoHs across 11 countries, serving the most vulnerable populations in rural and peri-urban communities. PIH supports the development of safe, effective, culturally sound, public mental health services within health system strengthening efforts. The PIH Cross-Site Mental Health Programme supports local care delivery capacity at each site through a transnational consultation model established on four pillars: sustained mentorship; programme implementation; nimble use of monitoring, evaluation and technology; and locally driven targeted research support (Partners In Health, 2020). Each PIH country site develops community-based mental health services that best fit their goals and context by establishing consensus on priority mental health conditions and treatment packages, while supporting human resource and management capacity building to implement effective mental health care pathways. Mental health care delivery is integrated into primary care and communities through “task-sharing”, enabling nonspecialist and lay providers to deliver care (Raviola et al., 2019). Across PIH sites, it was recognised that manualised, low-intensity, psychological interventions such as PM+ had potential to expand access to non-pharmacological services for common mental health conditions such as depression, stress and trauma-related conditions. We describe Rwanda’s adaptation in detail as the first PIH site to pilot PM+, with key examples from Peru, Mexico and Malawi to illustrate the cross-site adaptation process.

### Rwanda Adaptation Process

The MoH of Rwanda has decentralised mental health services from specialised facilities into primary care and communities as part of the national mental health policy since the 1994 genocide. However, mental health human and capital resources remain limited (Smith et al., 2020). PIH, known locally as Inshuti Mu Buzima (IMB), has supported public health system strengthening in three rural districts for 15 years. Following the successful integration of basic mental health service delivery for the most severe conditions into community and primary care settings in Burera district (Smith et al., 2017a,b), PIH/IMB identified the need to introduce a psychological intervention such as PM+ into its service delivery framework to address common mental health conditions.

Upon identifying the need for a psychological intervention, an effective process for intervention adaptation and implementation was articulated. Our approach reflects evidence-based recommendations for adapting psychological interventions to culture and context. Cultural adaptations include “systematic modifications of a psychological intervention that consider cultural patterns, meanings, and value to those who will receive the intervention”. Furthermore, contextual adaptation reflects considerations of the broader social, economic and political context of intervention recipients and the health system (Bernal & Sáez-Santiago, 2006; Bernal et al., 2009; Movsisyan et al., 2019). Elements of formal cultural and contextual adaptation frameworks were considered while attending to site needs for timely and practical service capacity building to address common mental health conditions. The key phases of our adaptation process are outlined in Box 1.

### Preparation for Adaptation

First, the PIH/IMB mental health teams conducted a literature review of peer-reviewed and gray literature on psychological interventions designed for delivery by nonspecialist providers. In late 2016, members of the local Rwandan MoH in Burera District, IMB and PIH reviewed findings and selected PM+ for individuals as it was: (1) transdiagnostic and scalable, (2) feasible for delivery in primary care and (3) had been tested for efficacy in the African context in Kenya (Bryant et al., 2017).

To facilitate the adaptation of PM+, PIH/IMB established an interdisciplinary Technical Working Group (TWG) comprised of existing staff and project stakeholders. The TWG included local and international psychiatrists, public health specialists, mental health programme coordinators, primary care nurses, a psychologist, a community health care manager, a curriculum development specialist and expert translators. The group requested WHO’s participant (trainee) and facilitator (trainer) materials from the World Vision team that conducted the prior PM+ effectiveness trial in Kenya. Local stakeholders and PM+ experts in other contexts were consulted about PM+ implementation, for example, through conference calls between the TWG and the Kenyan research team.

Incorporating MoH input to ensure alignment with the national mental health plan, the TWG selected the target population and cadres of healthcare providers who would implement PM+. The primary care setting was chosen to reach a broad population with common mental health conditions, focusing on depression. Primary care nurses were selected as providers because they already had basic mental health care delivery skills through previous mental health programme development and good interpersonal skills. A newly hired psychologist supervisor mentored the primary care nurses. Community health workers (CHWs) were identified to support case finding and social workers would help with community reintegration.

### Intervention Adaptation and Implementation Planning

First, the TWG reviewed the facilitator and participant manuals from World Vision and the original WHO versions. During a series of in-person and remote meetings between December 2016 and February 2017, the TWG identified content or language less suited to the Rwandan context that required adaptation, such as needing to change occupations in case studies from factory workers to rural farmers. Simultaneously, opportunities for contextualisation to Rwanda were noted, for example, adding background on the lasting mental health impact of the 1994 genocide. Lastly, opportunities to add content specific to routine care delivery were identified, such as the need to develop a PM+ care pathway for the Rwandan health system. Content referring specifically to implementing research studies was earmarked for removal.

After noting all potential changes, the TWG solicited feedback from additional implementers familiar with the target population and setting, such as the local IMB staff and the PM+ team from Kenya. Oversight of tasks and changes were managed by two project coordinators for organisation and communication using a project tracker.

The original WHO manual design was preserved. Adaptations focused on contextualising information specific to the care delivery setting, role-play adaptations, adding facilitator and participant prompts, language adaptation and implementation guidance. Key adaptations are summarised in Table 1. For example, language was added to the “managing problems” section to help nurses identify culturally relevant problems individuals could influence such as “the conflicts with my husband around paying children’s school fees”. The facilitator guide was also adapted based on cultural norms, names, idioms and phrases, and replacing text with images. For example, guidelines were adjusted to reflect Rwandan approaches to physical contact. Healthcare terminology was adapted such as changing “client” to “patient” and “helper” to “health centre nurse” as the term patient was already used in the primary care settings where PM+ would be implemented.

Materials were then adapted for routine care delivery. A depression clinical care pathway for stepped-care treatment based on symptom severity was adapted to include guidelines for pharmacological and non-pharmacological care, including PM+. Guidance for the management of individuals needing additional or alternate mental health care to PM+ was articulated, including emergency triage for acute crises, and exclusion criteria from the original PM+ research protocol were amended to reflect this. Guidance was also added to ensure PM+ delivery remained flexible to clinical needs exhibited by patients, including articulating possibilities for adapting session length and treatment duration (e.g. adding additional sessions), and for common primary care scenarios such as bringing family members to sessions. Locally validated clinical assessment tools designed to move people through specific care pathways (including the Patient Health Questionnaire (PHQ-9) for targeting depression) were prioritised for implementation rather than the full battery of assessment tools performed in the original PM+ research studies, given known time restraints of routine generalist nurses, and to prevent provider fatigue. Job aids, supervision and clinical tools were incorporated to support PM+ delivery quality and fidelity. For example, a clinical observation checklist was developed to help supervisors track the skills of lay providers delivering PM+, and for supervisors to use in providing feedback on clinical strengths and areas for provider improvement.

The training was reduced from 10 to 5 days followed by weekly on-site individual supervision for 6 months with a trained psychologist supervisor, given health centre nurses’ existing mental health background and practical time limitations on removing them from clinical responsibilities. As with the original PM+ manual, training methods included didactic lectures, case study analysis, role-plays and discussion. This adapted training included additional role-playtime to facilitate camaraderie and strategy practice.

Once the facilitator manual was complete, the TWG edited the participant manual and reconciled the two versions. The team prepared for PM+ training including adapting slides, training agendas and participant quizzes from the Kenya team’s materials. After materials were complete, content was reviewed for fidelity and translated by an external consultant into the local language, Kinyarwanda. The slides were also translated into French. Materials were back translated and reviewed in two stakeholder workshops by TWG members fluent in English, Kinyarwanda and French, who resolved discrepancies by consensus, and ensured local idioms were accurately described. All final products were printed and bound into training handbooks. To prepare for implementation in routine care, an electronic database was expanded to store supervision checklist data, and the electronic medical record used at primary care centres was updated for PM+ point of care data collection.

### Pilot Training, Testing and Revision

In March 2017, IMB conducted the first Training of Trainers (TOT) using the adapted PM+ materials with Burera District hospital-based mental health care providers including psychologist supervisors, psychiatric nurses and social workers. The TOT was designed to equip participants with the required knowledge to train primary health care nurses at health centres and to facilitate PM+ rollout. TOT content included the PM+ intervention and adult learning techniques. It was also an opportunity for providers to share experiences and provide feedback on the acceptability and feasibility of delivering PM+ in the local public primary health care system. Following the TOT, participants formed small discussion groups and provided further input on manual wording, training facilitation techniques, role-plays, provider skills gained, session flow, clinical factors and beneficiary response. Revisions incorporated all feedback and additional mentorship was recommended for providers to address knowledge gaps.

TOT participants then piloted and practised PM+ at the Burera District hospital outpatient mental health clinic from April 2017–August 2017 to ensure PM+ mastery. An expatriate psychiatrist provided supervision. Feedback was collected from community stakeholders and initial PM+ recipients revealing user satisfaction with PM+. In September 2017, a second training was conducted for PM+ providers, including additional MoH district-level supervisors, and two primary health nurses per health centre, followed by supervision for all participants. A rollout of PM+ across all health centres in the district was planned to occur over several years. Outcomes from the PM+ rollout process are forthcoming.

### PM+ Adaptation Across PIH Sites

Each PIH site planning to implement PM+ proceeded through the adaptation phases as outlined in Box 1. Sites sequentially started with materials already adapted by PIH instead of the general WHO versions to reduce duplication of efforts, reviewing the original WHO versions for comparison and to ensure completeness of relevant content. Table 1 summarises relevant site-level characteristics and key adaptations made to reflect local context.

### Socios En Salud/Peru

The Peruvian MoH began to decentralise mental health services in 2015, guided by WHO’s Mental Health Gap Action Programme, coordinating with nongovernmental organisations such as PIH Peru-Socios En Salud (SES). SES primarily supports a vulnerable peri-urban community in Carabayllo, Lima, recruiting and training CHWs and psychologists to identify and refer people with mental health conditions to government clinics, and to provide direct community-based interventions. SES supports a range of populations, including at-risk women and children (Eappen et al., 2018). The need to expand care for common mental health conditions became evident from observing the experiences of mothers of children participating in SES’ early childhood intervention programme, CASITA (Nelson et al., 2018).

SES integrated PM+ within their programmes for perinatal women. Bachelors-level psychologists were selected as PM+ providers. SES site leaders and PIH staff began the adaptation process by identifying and replacing all PIH Rwanda-specific information, including adaptations related to both culture and the health system. Training vignettes were edited to reflect types of adversity experienced by people living in peri-urban Lima, where PM+ would be implemented. Language was translated to Peruvian Spanish, and images were modified. Information directed to a medical audience in Rwanda (e.g. on psychotropic medication) was de-emphasised and replaced with information on psychiatric assessments by the MoH. In February 2018, a lead clinician from the PIH Cross-Site Mental Health Team and a SES postdoctoral fellow conducted 5 full days of in-person training for five SES psychologists. As with IMB, the training consisted of didactic presentations and discussion, role-plays and practice homework.

### Compañeros En Salud/Mexico

The Mexican MoH’s most recent National Health Programme includes guidelines on integrating mental health into primary care in community settings (Miguel-Esponda et al., 2020). PIH’s sister organisation in Mexico, Compañeros En Salud (CES), works closely with the MoH to provide access to high-quality services in marginalised communities across Chiapas, Mexico. Pasantes (young generalist physicians doing a government service year) provide mental health care in primary care clinics (Aguerrebere et al., 2019; Arrieta et al., 2017) and acompañantes (CHWs) offer psychoeducation, monitor treatment adherence and conduct community referrals. Historically, pasantes primarily offered psychotropic medicine with CHW follow-up for depression. However, the need to increase non-pharmacological services was identified.

Building on SES’s Latin American experience, in 2019 CES adapted PM+ to the Mexican context to address high rates of common mental health conditions in women, particularly in settings of gender-based violence. Guidance around the flexibility of the session length and number was added, particularly as providers found active listening beneficial for patients which often took longer than the time allotted in the 90-minute protocol. Furthermore, the WHO Psychological Outcomes Profile scale (PSYCHLOPS) was modified for literacy levels using a visual analogue mood scale instead of numbers. Five local community mental health workers (CMHWs) with elementary to high school education were hired to deliver PM+. Training preparation and adaptation were done in partnership with SES, contextualising Peru’s materials. SES colleagues travelled from Peru to Mexico to facilitate the PM+ training with CES for CMHWs in June 2019. A local psychologist was hired for weekly supervision, case management and CMHW continuing education. The existing depression care pathway was expanded for broader common mental health conditions addressed by PM+. PM+ was delivered by CMHWs at home visits, and participants were invited to join existing psychoeducation groups. To address high rates of trauma, separate training focused on grief, bereavement care, psychological first aid and trauma. This equipped CMHWs to provide trauma desensitisation sessions and/or violence safety planning for patients who disclosed traumatic experiences, before delivering PM+. Monthly staff meetings coordinated community- and facility-based care and provided interdisciplinary guidance on managing complex cases identified by CMHWs (Rodríguez-Cuevas et al., 2021).

### Abwenzi Pa Za Umoyo/Malawi

Abwenzi Pa Za Umoyo (APZU), PIH Malawi’s sister organisation in rural Neno district, has partnered with the Malawian MoH since 2007 including supporting the growth of mental health services. Malawi has some of the least available mental health funding, specialised human resource capacity and clinical services in Africa (Udedi, 2016). Mental health care supported by APZU is provided by mid-level nonspecialist clinical officers and nurses through the decentralised Integrated Chronic Care Clinic, combining human immunodeficiency virus (HIV), non-communicable diseases (NCD) and mental health services within APZU-supported primary health care facilities in Neno. An advanced mental health clinic at Neno District Hospital and Lisungwi Community Hospital provides support to patients with severe mental health conditions and epilepsy. However, there remains a paucity of counselling and care for people with common mental health conditions.

APZU began to adapt PM+ for the Malawian context in 2019. APZU was the first PIH site to implement group PM+ as it was innovative, cost-effective given limited providers, had the potential to reach a wider population and the peer support approach was deemed appropriate for the context. As the group PM+ manual was not yet publicly available at the time of adaptation, APZU obtained WHO approval to use the group model. The initial population APZU targeted for PM+ delivery was women in the perinatal period, due to the higher risk of depression during this time. This further informed the decision to implement group PM+, as group counselling has been shown to be effective for women with postpartum depression (Zlotnick et al., 2001). Three lay health counsellors with secondary education and one psychologist supervisor were hired. The Rwandan PM+ materials were contextually adapted in collaboration with IMB, referring to WHO manuals. An additional mental health background was added to the training materials for the lay counsellors, along with guidance on facilitating PM+ in group settings. The curriculum was adapted to address the target population, including case examples of women with postpartum depression, and content added regarding sexual, gender-based violence and abuse. In October 2019, the psychologist supervisor from the IMB team travelled to Malawi to assist with the PM+ training. Once completed, lay counsellors, supervised by the psychologist, began collaboration with antenatal and postpartum nurses at the health facility through community sensitisation and screening women for depression.

### Implementation Progress to Date

Following the adaptation steps, PM+ has been progressively implemented across the four country sites. Table 2 shows the number and cadre of people trained to deliver PM+ at each site, including local supervisors and healthcare workers. Table 3 summarises the number of individuals enrolled in PM+ across sites to date.

## Discussion

This work aimed to outline broad processes used across PIH sites to adapt and implement PM+ within real-world settings. Most literature describing the adaptation of psychological interventions to context takes place within research settings and rarely articulates the adaptation process for routine care delivery, as was done with local implementers and stakeholders within PIH supported public sector health systems. Although each site is unique, sites employed similar adaptation methods that have applicability across other settings. Key cross-site adaptations contextualised for implementation in local settings are summarised in Box 2. Lessons learned can contribute to easier PM+ adaptation in new contexts, which may lack the required financial and human resources that are often available with research studies.

### Lessons Learned

Training and curriculum materials can be contextualised based on cultural and implementation considerations while maintaining fidelity to core psychological elements of PM+, such as behavioural activation (Bryant et al., 2017). Across our PIH sites, we focused on targeted adaptations to optimise stakeholder and recipient acceptability, reduce barriers to care and increase local delivery capacity. A TWG of key stakeholders to review curriculum and training modules for cultural fit can help identify where key changes should be made.

Our adaptations predominately focused on enhancing fit for context within existing materials as opposed to changing core content, such as contextualising case study examples about behavioural activation and not the substantive strategy itself. This provided a framework for one site to learn from another’s adaptation, while maintaining the core components. For example, after the Rwandan team contextualised case studies, the Malawi team adapted those cases for perinatal depression. We also found curriculum and training should be adapted to the trainee background and skill level. For example, more general mental health background was included in Malawi, whereas in Rwanda, more clinical information was added for nurses based on their prior mental health care delivery experience. It is also important to note practical adaptation and translation can be a time-intensive process, as contextualisation is necessary for both training and implementation. Proximity to the field and target populations was fundamental for iterative adaptation, and service user feedback will continue being collected as part of our ongoing evaluation of PM+ rollout across sites. Sharing lessons learned together with other PM+ implementers and materials (such as slides, manuals, pre–post tests and tools) can expedite the adaptation process. Our adaptation period was shortened from approximately 1 year in Rwanda to 4 months in Mexico and Malawi due to cross-site collaborations.

Evidence-based psychotherapies will not only need cultural adaptation for use in any new context but also require health system adaptation considerations for real-world routine care delivery. Training should be active, participatory and iterative for feedback, with focused attention to ensure trainees demonstrate PM+ strategies adequately. For practical purposes, training time was shortened to 5 days at most sites with additional role-play time. Sustained supervision and mentorship following training were essential for providers to master skills, offer opportunities for ongoing discussion on complex cases and provide feedback for improvements to the initial intervention adaptation.

Though the original PM+ manual is comprehensive, additional guidance on PM+ implementation was needed, such as care pathways, supervision checklists and tools for ongoing care delivery. For example, though five sessions are standard in PM+, providers in Rwanda and Mexico found some flexibility in session length and number was useful, as patients often benefited from additional sessions and practice. Additionally, guidance on effective PM+ data collection methods and platforms were not provided in the initial PM+ intervention manual, thus all sites integrated PM+ information into ongoing data collection systems for symptom tracking, and to monitor quality of care and intervention uptake. For example, Rwanda integrated PM+ into the government health facilities’ electronic medical records system. Malawi developed an electronic patient satisfaction survey for completion after PM+, designed for using patient feedback to inform service delivery improvements.

Partnerships among local implementers, clinicians, supervisors, patients, communities and government were essential for understanding the delivery context, target populations and obtaining buy-in for PM+ delivery. Partnerships were also essential for iterative adaptation, pilot and testing processes. Site-to-site collaborations, such as between Mexico and Peru, provided opportunities for robust cultural exchanges and sharing lessons learned, which avoided recreating the wheel in material development. Ultimately, we found building capacity and integrating PM+ within existing public health services is feasible by selecting locally available cadres, which requires leadership and input of local communities and MoHs from the beginning.

### Limitations

Several limitations of the methodology should be acknowledged. The four settings described are part of a large non-governmental NGO with embedded cross-site capacity-building functions and long-standing relationships with local MoHs. Replication of the PM+ adaptation process in other settings may require additional coordination and partnership building early on. Formal evaluation is needed to determine if the adaptation processes ultimately resulted in effective PM+ implementation and to evaluate the clinical effectiveness of PM+ in routine settings. Furthermore, given the existing MoH and NGO infrastructure available in our settings, a cost-effectiveness study could help determine the feasibility of this process for replication and scale. Additional engagement and feedback from PM+ beneficiaries, providers and community members around perceptions of feasibility, acceptability and benefit would improve the cultural and contextual adaptation process for future field-level adaptations, implementation and scale.

## Conclusion and Future Directions

Our experience demonstrates PM+ is translatable across cultures and feasible for use in real-world public sector primary care and community contexts, outside of research and emergency response settings, in partnership with MoH. PM+ has the potential to be scaled nationally across new districts in Rwanda, Peru, Mexico and Malawi outside of PIH’s catchment areas using the locally adapted curriculum package. Furthermore, this adaption model can be replicated. For example, leveraging lessons learned from this process, early phases of PM+ adaptation are underway for delivery in the United States by a mobile outreach van in urban Boston and by social work students in Florida. With the emergence of the unprecedented COVID-19 pandemic, PM+ could be used across multiple platforms to support populations affected by the pandemic. Finally, there is potential for PM+ training to be delivered remotely on e-learning platforms. Adapting trainings from in-person to virtual will likely present its own set of unique implementation challenges.

Evaluation studies in Rwanda and Malawi and analyses of routine data in Mexico and Peru are underway to document and describe implementation pilot effectiveness and clinical outcomes of the contextualised PM+ intervention across these four settings.

## Figures and Tables

**Table 1:** Routine Care Delivery Model Informed Adaptations for Implementation (Site-Level Characteristics)

	Rwanda	Peru	Mexico	Malawi
**Location**	Rural	Peri-urban	Rural	Rural
**Model**	Individual	Individual	Individual	Group
**Language**	Kinyarwanda (some French) Changed “client” to “patient” and “helper” to “health centre nurse”	Spanish (Peruvian), Changed “client” to “participant”	Spanish, Changed “client” to “patient” and “helper” to “cuidadora” or “carer”	Chichewa
**Patient population**	People with common mental health conditions	Women with depression who are caregivers for children enrolled in an early-childhood intervention, Expanded for comorbid conditions (NCDs, COVID-19 and tuberculosis)	People with common mental health conditions	Women with perinatal depression
**PM+ providers**	Health Centre Nurses (primary care provider), CHWs (case identification), Social Workers (reintegration)	Psychologists	Community Mental Health Workers: *Cuidadoras*	Lay Counsellors
**Trainers**	PIH Cross-Site Mental Health Team and Rwandan supervisors	PIH Cross-Site Mental Health Team and Peruvian leadership	Peruvian and CES leadership	Rwandan Psychologist
**Supervision and mentorship**	Psychologist and Psychiatric Nurse	Psychologist	Psychologist and Mental Health Coordinator (physician)	Psychologist
**Health system level**	Community (for behavioural activation), Health Centre District Hospital	Community	Community	Community, Health Centre
**Number of clinics or communities**	19 health centres and 1 district hospital	4	5	1

Note. For access to training materials or tools, please contact xsitementalhealth@pih.org. CES, Compañeros En Salud, CHW, Community Health Worker.

**Table 2:** Number of People Trained to Deliver PM+ Across PIH sites

Country	CHW or lay counsellor	Psychologist or social worker	Nurse	Supervisors	Total
Rwanda	225	4	65*	11	305
Peru	4	30*	–	–	34
Mexico	6*	1	–	1	8
Malawi	3*	1	–	–	4
Total	238	36	65	12	351

* Note. Primary implementers for PM+ delivery. As PM+ was sequentially adapted across sites, the time period varies by location. Data reflect the following dates: Rwanda: March 2017–June 2020; Peru: January 2018–June 2020; Mexico: June 2019–June 2020; and Malawi: October 2019–June 2020. CHW, Community Health Worker; PIH, Partners in Health; PM+, Problem Management Plus.

**Table 3:** Number of People Newly Enrolled into PM+ Across PIH Sites

Country	Year				
	2017	2018	2019	2020 (January–June)	Total
Rwanda	17	176	523	297	1013
Peru	–	45	50	165	260
Mexico	–	–	50	20	70
Malawi	–	–	–	18	18
Total	17	221	623	500	1361

Note. The increase in patient numbers per year is attributed to progressive roll out across health centres in Rwanda. A decrease in patients in 2020 is attributed to service delivery interruptions due to COVID-19. PM+, Problem Management Plus, PIH, Partners In Health.

## Box 1: Key Phases to Adapt PM+ to Context

(1) Preparation for Adaptation
  - Conducted literature review and selected PM+
  - Established a PM+ adaptation Technical Working Group
  - Selected target population and health worker cadre to deliver PM+
  - Consulted local stakeholders, government and implementers
  - Consulted PM+ experts in other contexts
(2) Intervention Adaptation and Implementation Planning
  - Identified areas for content and delivery modifications needed through comprehensive Technical Working Group review, including:
    (1) Cultural modification for language, case studies, images and idioms
    (2) Implementation modifications to align with the health system context
  - Consulted local stakeholders to inform modifications
  - Iteratively revised manuals within the Technical Working Group
  - Adapted training length and content complexity for provider level
  - Incorporated additional role-plays, practical skill-building and active training methods
  - Developed care pathway for PM+ use within the health system, including adapting assessment protocols for routine care
  - Attained local and expert stakeholder feedback on manual revisions, care pathway and other materials
  - Translated to local language
(3) Pilot Training, Testing and Revision
  - Completed a Training of Trainers
  - Obtained and incorporated feedback following the Training of Trainers
  - Piloted PM+ implementation locally
  - Conducted field observations and piloted supervision, including creating and/or adapting existing supervision materials
  - Incorporated feedback from community stakeholders and PM+ recipients
  - Planned for expanded training and intervention rollout

## Box 2: Key Cross-Site Adaptations

- Cultural and implementation modifications contextualised to setting
- Intervention protocols adapted based on individual or group PM+
- Contextualised case studies, material and patient profiles in vulnerable communities
- Tailored active training methods to provider cadre and knowledge level
- Adapted language to local context, literacy level and translated materials
- Supervisors trained and maintained ongoing supervision with providers
- Iteratively adapted protocols for PM+ integration into routine care through pilot

## Key implications for practice

- PM+ can be contextualised based on cultural and implementation considerations while maintaining core psychological elements across different settings.
- The adaptation of PM+ for local health systems and articulation of practical guidance on implementation for routine care is essential.
- Partnerships across implementing sites are fundamental for iterative PM+ adaptation and provide opportunities for sharing lessons learned.
